# Four distinct types of dehydration stress memory genes in *Arabidopsis thaliana*

**DOI:** 10.1186/1471-2229-13-229

**Published:** 2013-12-30

**Authors:** Yong Ding, Ning Liu, Laetitia Virlouvet, Jean-Jack Riethoven, Michael Fromm, Zoya Avramova

**Affiliations:** 1 University of Science & Technology of China, 443 Huangshang Road, Hefei, Anhui 230027, China; 2 University of Nebraska School of Biological Sciences, 1901 Vine Street, Lincoln 68588, USA; 3 University of Nebraska Center for Biotechnology and Center for Plant Science Innovation, 1901 Vine Street, Lincoln 68588, USA

## Abstract

**Background:**

How plants respond to dehydration stress has been extensively researched. However, how plants respond to multiple consecutive stresses is virtually unknown. Pre-exposure to various abiotic stresses (including dehydration) may alter plants’ subsequent responses by improving resistance to future exposures. These observations have led to the concept of ‘stress memory’ implying that during subsequent exposures plants provide responses that are different from those during their first encounter with the stress. Genes that provide altered responses in a subsequent stress define the ‘memory genes’ category; genes responding similarly to each stress form the ‘non-memory’ category.

**Results:**

Using a genome-wide RNA-Seq approach we determine the transcriptional responses of Arabidopsis plants that have experienced multiple exposures to dehydration stress and compare them with the transcriptional behavior of plants encountering the stress for the first time. The major contribution of this study is the revealed existence of four distinct, previously unknown, transcription memory response patterns of dehydration stress genes in *A.thaliana.* The biological relevance for each of the four memory types is considered in the context of four overlapping strategies employed by a plant to improve its stress tolerance and/or survival: 1) increased synthesis of protective, damage-repairing, and detoxifying functions; 2) coordinating photosynthesis and growth under repetitive stress; 3) re-adjusting osmotic and ionic equilibrium to maintain homeostasis; and 4) re-adjusting interactions between dehydration and other stress/hormone regulated pathways.

**Conclusions:**

The results reveal the unknown, hitherto, existence of four distinct transcription memory response types in a plant and provide genome-wide characterization of memory and non-memory dehydration stress response genes in *A.thaliana*. The transcriptional responses during repeated exposures to stress are different from known responses occurring during a single exposure. GO analyses of encoded proteins suggested implications for the cellular/organismal protective, adaptive, and survival functions encoded by the memory genes. The results add a new dimension to our understanding of plants’ responses to dehydration stress and to current models for interactions between different signaling systems when adjusting to repeated spells of water deficits.

## Background

Environmental stresses exert evolutionary pressure on organisms, which, in turn, have developed sophisticated responses to cope and to survive. Plants react to unfavorable conditions by dynamically changing physiological behavior and expression levels of implicated genes. Pre-exposure to various abiotic stresses (salinity, cold, high temperature) may alter plants’ subsequent responses by improving resistance to future exposures [1,2]. Pre-treatment (priming) with hormones (jasmonic acid, JA, abscisic acid, ABA, salicylic acid, SA) increased the systemic immunity and induced stronger responses from responding genes upon subsequent treatments, relative to non-primed plants [3-7]. These observations have led to the concept of ‘stress memory’, implying that during subsequent exposures plants provide responses that are different from their responses during the first encounter with the stress [8].

Drought-triggered dehydration stress is one of the most common environmental stresses endured by plants and their responses to dehydration stress are extensively researched at organismal, cellular, and genome levels. Recently, we demonstrated that Arabidopsis plants subjected to several cycles of dehydration/water recovery treatments maintained higher relative water content than plants experiencing dehydration stress for the first time [9]. Dehydration stress ‘memory’ also affected gene expression from response genes. Analysis of 14 dehydration response genes after multiple stress treatments revealed the existence of two different types of transcriptional responses: genes that produced transcripts at similar levels during each stress and genes that in a subsequent response significantly increased their transcript levels and transcription rates relative to the first response [9]. Accordingly, genes that provide altered responses in a subsequent stress were referred to as ‘memory genes’ to distinguish them from ‘non-memory’ genes responding similarly to each stress.

Here, we explore how broadly memory genes are distributed within the dehydration stress responsive fraction of the *Arabidopsis thaliana* genome. Comprehensive RNA-Seq analyses of the transcriptomes of plants prior to stress, during a first stress, and during a third dehydration stress exposure revealed an unexpected diversity of memory-type responses at the transcriptional level. Analyses of the memory genes by GO functional categories indicated a biased functional distribution among the memory response patterns. Results are interpreted in the context of the possible biological relevance of these memory responses.

## Methods

### Plant growth and treatments

*Arabidopsis thaliana* (Col-0) plants were grown in potting soil in growth rooms at 22°C with a 12-h light photoperiod and light intensity of 180 μmol m^-2^ s^-1^. Repeated dehydration stresses were performed by air-drying for 2 h followed by a 22 h period of full re-hydration recovery as described [9]. RNA-Seq analyses were performed on rosette leaves from pre-stressed (W) plants, from plants exposed to the first dehydration treatment (S1), and during a third stress (S3) following two stress/recovery cycles. Plants from two independent biological samples were used. After removal from soil and before initiating the stress/recovery cycle, experimental plants were conditioned overnight in humid chambers and used to establish the basal transcript levels (W) for the transcriptome analyses. The transcriptional behavior of the marker memory genes *RD29B* and *RAB18* were monitored in leaf samples taken from fresh plants in soil, from overnight conditioned plants after their removal from soil, and from plants kept under watered conditions throughout the stress/recovery as indicators of the normal dehydration stress behavior of plants in S1 and S2 and internal controls for the whole genome transcriptome analyses.

### RNA extraction and RNA-Seq library construction

Leaf tissues were collected and immediately frozen in liquid nitrogen. Total RNA was extracted with Trizol (Invitrogen Inc. Carlsbad, CA, USA), treated with DNase I (Qiagen, Valencia, CA), and purified using Qiagen RNeasy Mini Kit. RNA integrity was confirmed on a Bioanalyzer 2100 using Nano 6000 LabChip (Agilent Technologies, Santa Clara, CA). Complementary DNA sequencing library was prepared from the total RNA using the mRNA-Seq Sample Preparation Kit (Illumina, San Diego, CA). Briefly, poly-adenylated RNA was isolated from 10 μg total RNA by Sera-Mag Magnetic Oligo-dT beads (ThermoFisher Scientific, Waltham, MA). RNA Purified mRNA was fragmented, annealed to high concentrations of random hexamers, and reverse transcribed. Following second strand cDNA synthesis, end repair, and A-tailing, Oligo adapters complementary to sequencing primers were ligated to cDNA fragment ends. Resultant cDNA libraries were size fractionated on an agarose gel, 200 bp fragments excised, and amplified by 15 cycles of polymerase chain reaction. Clusters were generated from the cDNA sequencing library on the surface of a flowcell in the Cluster Station (Illumina) by so-called bridge amplification. Replicates for the watered, S1 and S3 sample libraries were each run on a single lane in a flowcell on an Illumina GAIIx at the Genomics Core Facility at the University of Nebraska-Lincoln.

### Reverse transcription and real-time PCR

Total RNA isolation and reverse transcription with oligo(dT) (18418–012, Invitrogen) were performed as described previously [10]. The amounts of individual genes were measured with gene-specific primers by real-time PCR analysis with a CyclerIQ real-time PCR Instrument (Bio-Rad) and SYBR Green mixture (Bio-Rad). The relative expression or amount of specific genes was quantitated with the 2^-ΔΔ*C*t^ calculation [11], according to the manufacturer’s software (Bio-Rad), where the reference gene was ubiquitin. Primers used in real-time RT-PCR are in Additional file 1: Table S1.

### Bioinformatics analysis

Transcriptome sequencing of the watered, S1, and S3 samples yielded a total of 53.4, 76.8, and 78.4 million reads, respectively, summed over the two biological replicates per sample (see Additional file 2: Table S2). The read length for S1 and S3 is 75 bases, while for watered it is 101 bases. To determine the quality of the replicates we performed a least-square simple linear regression for each of the three samples. We calculated the *R*^*2*^ statistic (0.96 ≤ *R*^*2*^ ≤ 0.99) and slope (1.07 ≤ *b* ≤ 1.09), which provide measures of goodness-of-fit and correlation, respectively, using the *regress* function in MATLAB® (version 8.0.0.783 [R2012b]; The MathWorks™) (Additional file 3: Table S3). For use in all further analyses, the *Arabidopsis thaliana* genome and gene models were downloaded from Illumina’s iGenomes (Ensembl, TAIR10), and gene functional descriptions and Gene Ontology assignments from the Arabidopsis Information Resource (release TAIR10, [12]).

The bowtie (version 2.1.0; [13]) and tophat (version 2.0.8; [14]) packages were used with default parameters to map the RNA sequence reads from watered, S1, and S3 to the genome (see Additional file 3: Table S3), and to determine the expression quantity of known transcripts in each sample. The *cuffdiff* tool from the cufflinks package (version 2.0.2; [15]) was run with default parameters to calculate expression changes and associated q-values (False Discovery Rate adjusted p-values) for each gene, between the samples S1 and water, and S3 and S1. We further classify genes as being significantly differentially expressed when all three of the following conditions are met: *q* ≤ 0.05; | log_2_(fold change) | ≥ 1; and the FPKM-normalized expression value of at least one sample out of the two needs to be larger than the 25^th^ percentile. The output files of *cuffdiff* are further annotated (in-house Perl script) by adding gene functional descriptions and GO classifications, and merged into a master file containing all data for S1 versus water and S3 versus S1 (Additional file 3: Table S3, Additional file 4: Table S4, and Additional file 5: Table S5).

From that master file we determined the 6579 significant drought-responsive genes (S1 versus water), and using that initial set we then looked at the significant responses in S3 versus S1 (1963 genes) and the remainder that did not respond (4616 genes). We assigned simple classifications to the types of response during the first stress (+ or -) and the second stress (+, -, or =), combining them into six classes: [++], [--], [+-], [-+], [+=], and [-=] (Table 1; Table 2; Additional file 4: Table S4, Additional file 5: Table S5). Two additional classes [=/+] and [=/-] contain genes that in S1 did not change significantly expression (according to the three criteria for significance, above) compared to pre-stressed levels in W but significantly changed transcription in S2. Formally, these genes do not belong to the S1 dehydration-stress responding fraction.

**Table 1:** **Dehydrations response and memory genes in *Arabidopsis thaliana***

		**Total**	**Up**	**Down**
Total genes		33,555		
Dehydration response		6,579	3396	3183
Memory genes		1963	1219	744
	[+/+] W < S1 < S3	362		
	[-/-] W > S1 > S3	310		
	[+/-] W < S1 > S3	857		
	[-/+] W > S1 < S3	434		
Non-memory genes				
	[+/=] W < S1 = S3	2,177		
	[-/=] W > S1 = S3	2,439		
Late-response genes				
	[=/+] W = S1 < S3	798		
	[=/-] W = S1 > S3	573		

**Table 2:** Distribution of dehydration stress responding genes according to GO function

		**[+/+]**	**[-/-]**	**[+/-]**	**[-/+]**	**[+/=]**	**[-/=]**	**[=/+]**	**[=/-]**
		**362**	**310**	**857**	**434**	**2177**	**2439**	**798**	**573**
Ribosomal and protein synthesis		1	31(10%)	1	0	10 (0.5%)	135 (5%)	2	26 (5%)
Response to salt		25 (7%)	8 (3%)	84 (10%)	14 (3%)	154 (7%)	55 (2%)	35 (4%)	24 (4%)
Response to cold/heat		28 (8%)	18 (8%)	53 (6%)	19 (4%)	147 (7%)	72 (3%)	40 (5%)	46 (7%)
Response to ABA		24 (7%)	6 (2%)	80 (9%)	12 (1%)	143 (7%)	38 (1%)	39 (5%)	24 (4%)
LEA		12 (3%)	0	4	2	9	2	5 (0.7%)	0
Response to light		18 (5%)	25 (8%)	28 (3%)	24(6%)	68 (3%)	101 (4%)	47 (6%)	27 (5%)
	UV	3	2	4	5	10	18	12	6
	Intensity	11	10	13	11	40	66	27	14
	Red/far red	2	12	7	3	14	16	6	7
	Blue	2	1	3	5	2	10	2	0
Circadian rhythm		1	1	13	6	11	23	8	12
Chloroplast		12 (3%)	75 (24%)	20 (2%)	22 (5%)	48 (2%)	203 (8%)	26 (3%)	75 (15%)
Thylakoid membrane		0	53 (17%)	6 (0.7%)	9 (2%)	14	177 (7%)	7 (1%)	22 (4%)
Membrane		30 (8%)	23 (7%)	106 (12%)	41 (10%)	312 (14%)	233 (10%)	73 (9%)	71 (14%)
TM transport, porins		20 (5%)	6 (2%)	62 (7%)	26 (5%)	138 (7%)	95 (4%)	50 (6%)	24 (4%)
Wall/PM		13 (3%)	25 (8%)	30 (3%)	22 (5%)	48 (2%)	129 (5%)	38 (4%)	37 (6%)
Kinases, recepors, signal trans-duction		4	16 (5%)	52 (6%)	31 (7%)	107 (5%)	111 (5%)	25 (3%)	21 (4%)
Response to auxin		9 (2%)	5 (2%)	43 (4%)	10 (1%)	66 (3%)	48 (2%)	12 (1%)	12 (2%)
Response to ethylene		4 (1%)	3 (1%)	53 (6%)	8 (2%)	87 (4%)	24 (1%)	12 (%)	8 (1%)
Response to GA		2 (1%)	5 (2%)	10 (1%)	7 (1%)	13	18 (0.7%)	13 (1%)	5 (1%)
Response to JA		7 (2%)	16 (5%)	121 (14%)	19 (2%)	89 (4%)	46 (2%)	13 (1%)	21 (4%)
Response to SA		4 (1%)	5 (2%)	41 (5%)	13 (3%)	63 (3%)	24 (1%)	14 (1%)	12 (2%)
Transcription factors		29 (8%)	6 (2%)	73 (7%)	25 (6%)	161 (7%)	100 (4%)	51(2%)	22 (4%)
	AP2/ERF	5	1	16	2	19	10	1	3
	bHLH	2	1	16	2	11	16	5	0
	homeo_ZIP	3	1	6	3	25	16	8	3
	MYB	4	1	8	3	16	9	10	4
	ZF	3	2	6	11	41	28	12	4
	B_ZIP	4	0	4	2	13	1	3	0
	NAC	1	0	7	1	13	1	3	0
	GRAS	1	0	2	0	3	6	1	2
	HSF	1	0	3	0	4	0	2	1
	CCAAT	3	0	0	0	2	3	2	0
	WRKY	2	0	5	1	14	4	1	1

Number of genes and percentages per memory group are reported. Only percentages higher than 0.7 are reported; higher than 1% are rounded to the nearest integer.

The raw transcriptome sequence files for watered, S1, and S3 have been uploaded, together with gene expression result files, to NCBI’s Gene Expression Omnibus under sequence number GSE48235.

## Results

### Dehydration stress memory response genes of *Arabidopsis thaliana*

Watered non-stressed plants and plants subjected to one or three dehydration stresses by exposure to dry air for 2 hours followed by 22 h periods of watered recovery intervals [9] were analyzed. The levels of mRNAs from the leaves of these plants were determined by RNA-Seq. Transcripts from 33,555 *A. thaliana* genes were identified (Table 1; Additional file 3: Table S3)*.* Of these, 6579 genes (~20% of the genes in the genome) significantly increased or decreased their transcript levels during the first dehydration stress (S1), compared to their transcript levels in watered plants (W), representing the dehydration stress-responsive genes of *A. thaliana.* Of the genes responding in S1, 3396 genes were up-regulated and 3183 were down-regulated (Table 1; Additional file 4: Table S4)*.* A comparison of the transcript levels during the first (S1) and third dehydration stress (S3) identified 1,963 dehydration responsive genes displaying significantly different amounts of transcripts in S3 versus S1. Our operational criterion for transcriptional memory is that the transcriptional responses to similar stress conditions must be different. Accordingly, these 1,963 genes display dehydration stress transcriptional memory (Table 1; Additional file 4: Table S4).

Four distinct transcriptional memory response-patterns were recognized within the S1-responding fraction. We designate these transcription patterns as [+/+], [-/-], [+/-] and [-/+] memory responses, where the first sign indicates higher (+) or lower (-) transcript levels in S1 relative to the levels in pre-stressed watered plants (W). The second sign indicates higher (+) or lower (-) transcripts in S3 relative to the levels in S1. Non-memory genes are denoted as [+/=] or [-/=] indicating that up-regulated or down-regulated transcript levels occurring in S1, respectively, have similar levels in S3. In addition, 798 genes were up-regulated in S2 ([**=/**+] W = S1 < S3) and 573 were down-regulated ([**=/**-] W = S1 > S3). These delayed response patterns are different from the memory responses represented by the four S1-responding memory types. Here, we focus mainly on the transcriptional patterns of the S1-responding gene fraction of Arabidopsis.

**Figure 1:**
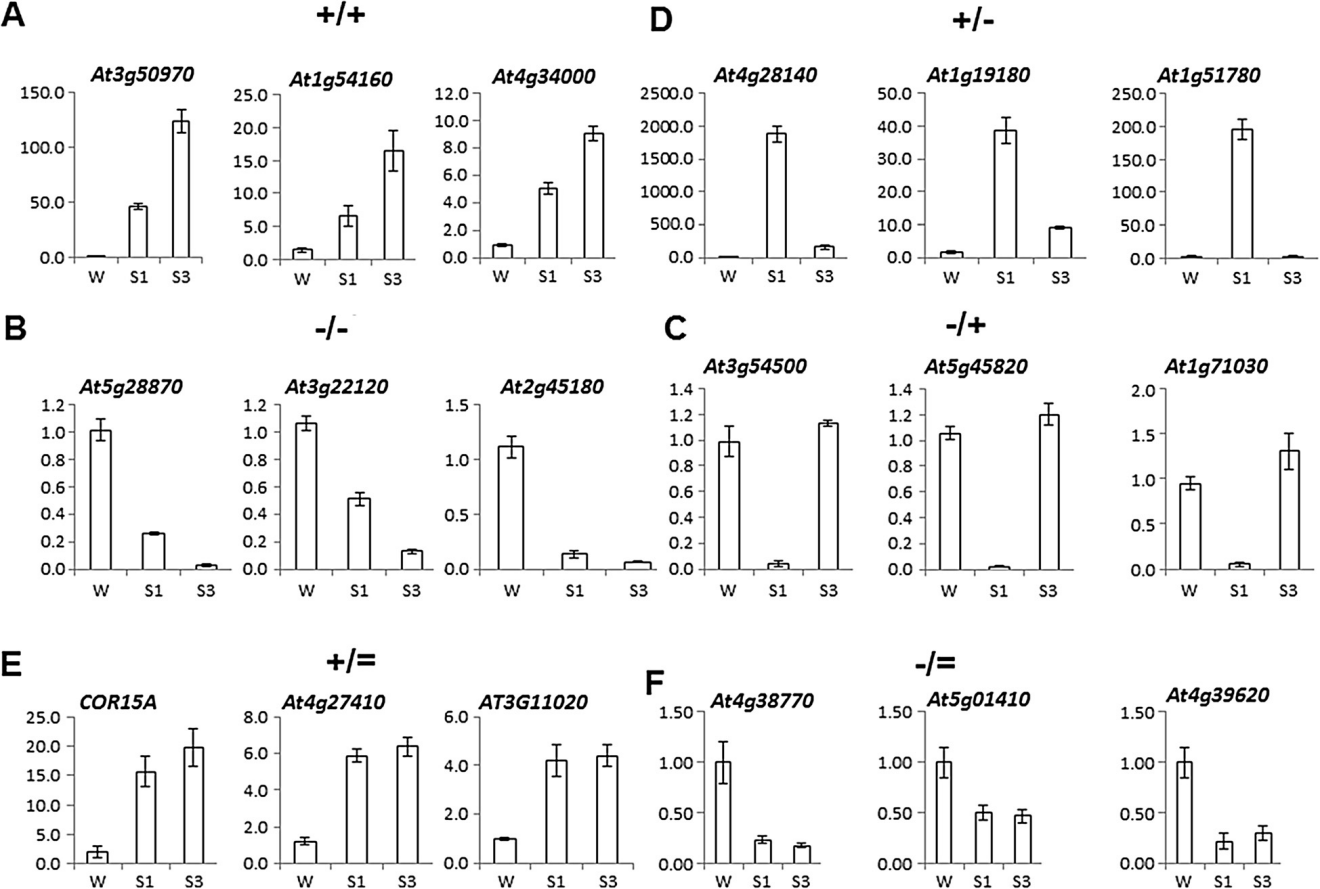
**Transcription memory response patterns of dehydration stress responding genes.** Three genes from each memory category were randomly chosen from the RNA-Seq datasets to illustrate the diverse transcriptional response patterns. **A)** [+/+] memory genes; **B)** [-/-] memory genes; **C)** [-/+] memory genes; **D)** [+/-] memory genes; **E)** [+/=] non-memory genes; **F)** [-/=] non-memory genes. Transcript levels measured by real-time qRT-PCR are under initial pre-stressed watered (W) conditions, after the first (S1), and the third (S3) exposures to dehydration stress. Measurements were performed on three biological samples, each with three technical replicates. Bars are the mean ± s.e.m., *n* = 3 replicates. For interpretation of the response signs see text.

**Figure 2:**
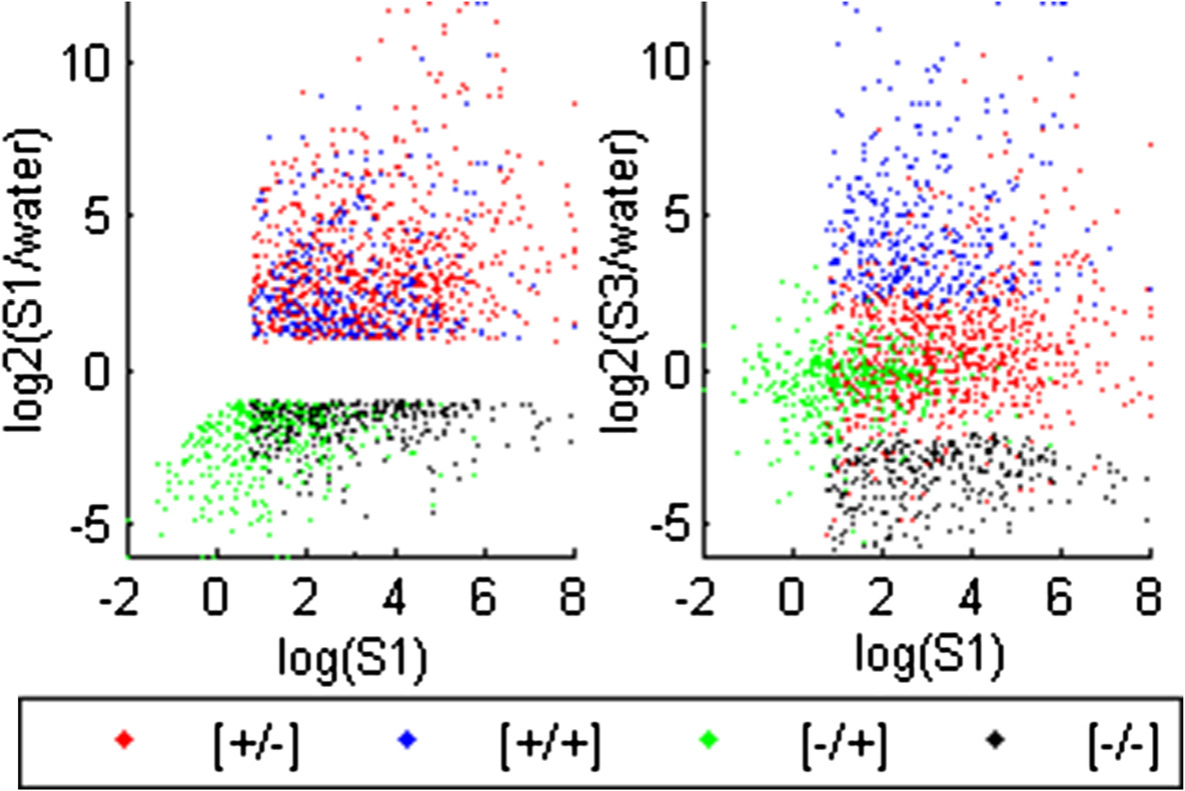
**Distribution of dehydration stress responding genes in *A. thaliana *in S1 and S3. Left panel).** Transcript levels from dehydration stress responding genes that are up-regulated or down-regulated during S1 (color key at the bottom) plotted by the log2 of their S1 levels along the x-axis, and by log2 of their S1/watered ratio along the y-axis. **Right panel**) Transcript levels of dehydration stress memory genes: as in (A) except the y-axis is the log2 of the S3/watered ratio. The clustering of the four colors illustrates the distribution of the four distinct memory response types: revised response [+/-] and [-/+] memory genes clustering closer to their pre-stressed (W) levels, while the [+/+] and [-/-] increasing separation from these levels.

Of the four different memory classes, 362 genes denoted as [+/+] memory genes were induced in S1 and induced to higher levels in S3 (Table 1; Figure 1A). All 7 memory genes described in our earlier study [9] were present in the [+/+] memory category in the genome-wide RNA-Seq data (Additional file 4: Table S4). The [-/-] class contained 310 genes with decreased transcript levels in S1 and further reduced levels in S3 (Table 1; Figure 1B). The [-/+] class contained 434 memory genes that were down-regulated in S1, but produced significantly higher transcript levels in S3 (Figure 1C); conversely, 857 [+/-] memory genes, were induced in S1 but had lower levels in S3 (Figure 1D). The latter two categories ‘revise’ their transcriptional behavior in a subsequent stress: after robustly responding in S1, these genes show weaker/no responses in S3, producing transcripts at levels close to their initial watered (pre-stressed) levels. These genes are referred to as ‘revised response’ memory genes. The distribution of transcript levels for the dehydration stress-responsive memory genes in S1 and S3 illustrating the four memory categories is shown (Figure 2A, B). The two non-memory gene classes ([+/=] and [-/=]) consistently provide transcriptional responses in S3 similar to those in S1 (Figure 1E, F).

### Functional distribution of Arabidopsis memory genes

The large number of the dehydration stress-memory responsive genes and the diversity of their transcriptional responses raised questions of their functions and of whether there was a preferential association of particular cellular functions with any of the four memory types. To address these questions, genes displaying transcriptional memory were analyzed for biological function according to their Gene Ontology (GO) classification (Table 2; Additional file 5: Table S5).

### Genes involved in ABA/abiotic stresses responses are signature for the [+/+] memory genes

**Figure 3:**
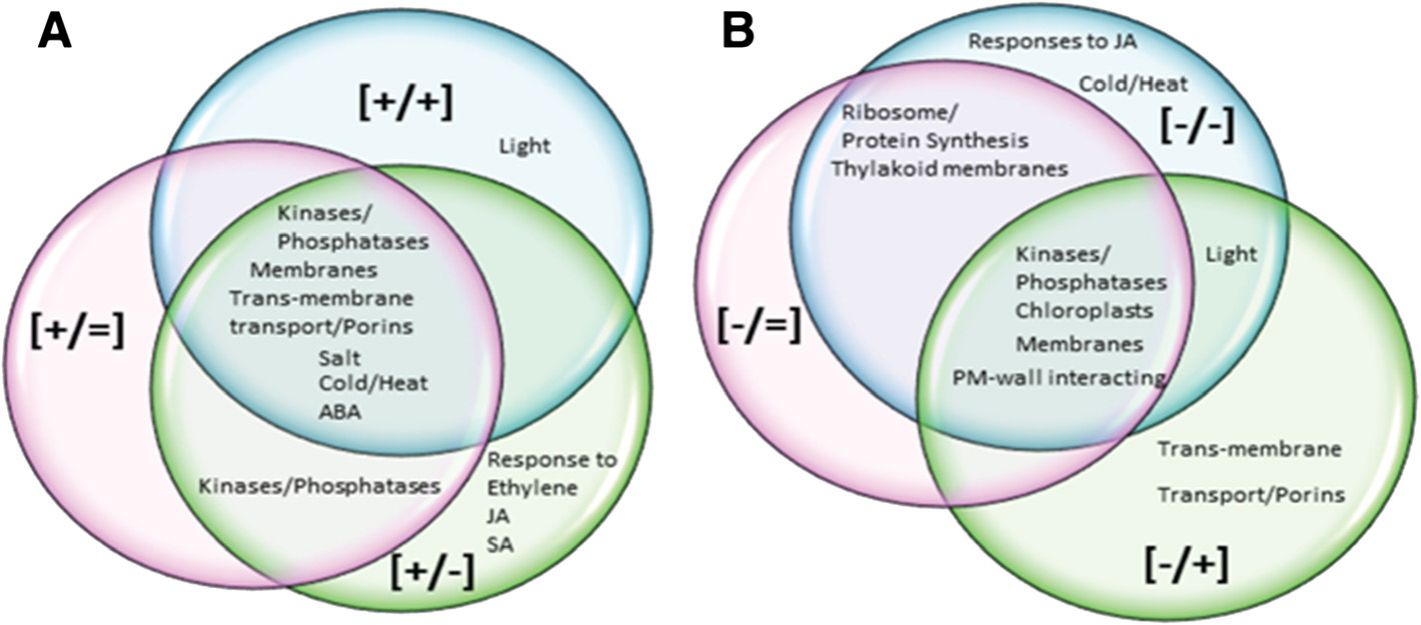
**Distribution of dehydration stress memory genes in *A. thaliana *according to GO functions.** The functional GO categories represented by the more abundant (5% or more of the genes within a memory response type) GO groups of responding genes. The four memory and two non-memory types are shown in a Venn diagram format: **A)** the two memory and one non-memory types up-regulated in S1; **B)** the two memory and one non-memory types down-regulated in S1.

Genes implicated in responses to salt, salinity, cold/heat acclimation, and abscisic acid (ABA) constitute about a quarter of the [+/+] memory genes. *LEA* genes represent 3% among them (Table 2; Additional file 5: Table S5; Figure 3).

### Ribosome/protein synthesis, chloroplast, and thylakoid membrane associated functions are signature for [-/-] memory genes

Memory genes encoding ribosomal, as well as chloroplast and photosynthesis proteins are the major constituents of the [-/-] memory subgroup (Additional file 4: Table S4; Additional file 5: Table S5; Figure 3). Encoded proteins are involved in ribosome structure and amino acid biosynthesis, as well as in photosynthesis, in the light harvesting complex (photosystem II), and in responses to light (red/far red light, in particular). The largest number of thylakoid membrane-associated proteins is encoded by [-/-] memory genes.

### Functions encoded by [-/+] revised response memory genes

There are no particular types of cellular functions specifically enriched within the [-/+] group of memory genes, although the largest percentages of genes encode chloroplast and membrane (plasma, organellar, and thylakoid membrane) related functions. Many of these are for electron transport, photosystem II assembly, chloroplast organization and re-location, functions similar to those encoded by [-/-] memory genes (Table 2; Additional file 5: Table S5; Figure 3).

### Dehydration stress [+/-] memory genes are shared with multiple signaling pathways

The signature [+/-] dehydration memory genes are regulated by multiple signaling pathways, including the ABA, ethylene, auxin (IAA), gibberrellic acid (GA), jasmonic acid (JA), and salicylic acid (SA) pathways (Table 2; Additional file 5: Table S5; Figure 3). A prominent functional group represented by [+/-] memory genes encodes proteins associated with membranes. Although membrane (plasma and organellar)-related genes are highly represented within the entire drought-responding fraction (including the non-memory and all four memory types), genes for tonoplast intrinsic proteins regulating water transport (GAMMA-TIP, PIP2B, TMP-C, PIP2A, TIP2, PIP1A, RD28) and the inward K^+^ channel proteins (KAT1 and KAT2) display, exclusively, [+/-] memory responses (Table 2; Additional file 5: Table S5).

### Dehydration stress memory of transcription factor genes

Among the ~1500 Arabidopsis genes encoding transcription factors (TFs) [16], members of about two dozen families have been implicated in responses to drought [17-19]. Among these families, transcriptional memory behavior was found for members of ten of the families (Additional file 6: Table S6). Five families (AP2/ERF, bHLH, Homeodomain-Zip, MYB/Myb-like, and ZF) are represented in all memory categories; GRAS and HSF members display only [+/+] and [+/-] memory, and the CAAT family is represented only by [+/+] memory genes. TF genes are least represented in the [-/-] memory subgroup (Table 2; Additional file 6: Table S6).

**Figure 4:**
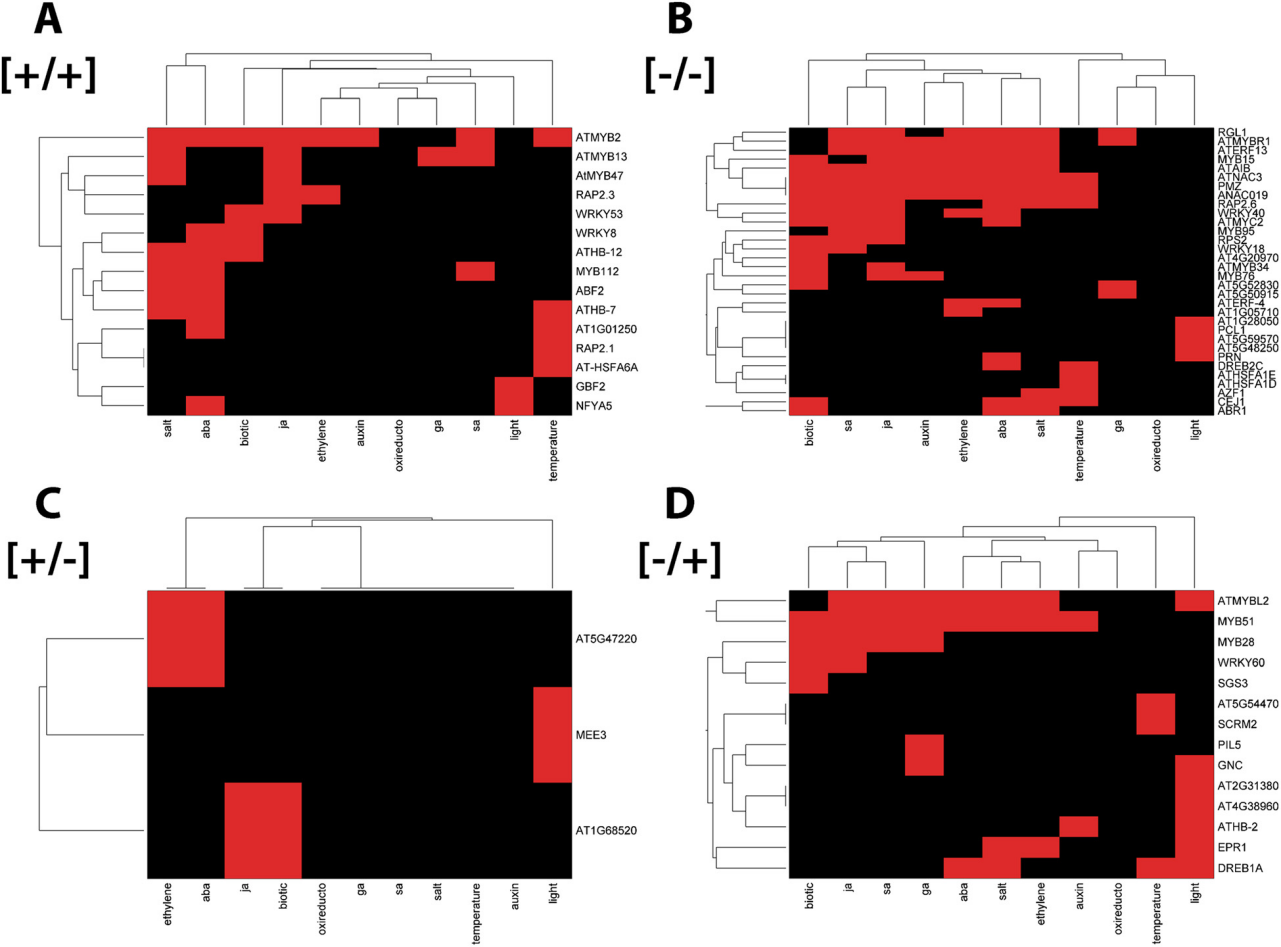
**Dehydration stress memory genes encoding TFs.** Hierarchical clustering of dehydration stress memory genes encoding transcription factors implicated in the crosstalk between various abiotic stress and hormone regulated pathways. Presence (red, value 1) or non-presence (black, value 0) of annotation in stress and hormone regulated pathways is determined for each transcription factor, and then clustered along both rows (genes) and columns (annotation) via unweighted average Euclidean distance. **A)** TF genes displaying [+/+] memory; **B)** [-/-]; **C** [+/-]; **D** [-/+].

[+/+] memory genes for MYB2, MYB112, MYB13, MYB47, ATHB-7, ATHB-14, and ABF2 specifically cluster with ABA/abiotic stress responding genes but are rarely found among other hormonally responsive pathways (Figure 4A); six putative TF genes display [-/-] memory type responses and only three are shared with other response pathways (Figure 4B). TFs with [+/-] or [-/+] revised response memory behavior are broadly shared with multiple abiotic and hormone response networks, outlining distinct clusters (Figure 4C, D).

## Discussion

### Biological relevance of the transcription memory behavior

Plants experience more dehydration stress during the day when transpiration exceeds the rate of water uptake and fully, or partially, recover during the night when the rate of water uptake exceeds the greatly diminished loss of water from leaves [20]. This creates a diurnal cycle of oscillating leaf water potential that is probably most pronounced during periods of moderate drought conditions [21,22]. The repetitive dehydration stress system employed in this study has similarities to this natural diurnal stress. We hypothesize that dehydration stress memory helps plants to prepare for the next day’s stress if they were stressed the day before, despite alleviated stress signals during the night [9].

Genes displaying transcriptional memory are among the genes responding to the first dehydration stress. Altering their expression levels in subsequent stresses, presumably, allows the plant to finely tune its responses to ongoing/recurring dehydration stress. The possible biological relevance of the genes displaying transcriptional memory is considered in the context of four overlapping strategies generally employed by a plant to improve its stress tolerance and/or survival: 1) increased synthesis of membrane protecting, damage-repairing, and detoxifying functions; 2) coordinating photosynthesis and growth under repetitive stress; 3) re-adjusting osmotic and ionic equilibrium to maintain homeostasis [23]; and 4) re-adjusting interactions between dehydration and other stress/hormone regulated pathways [24,25].

### Increased synthesis of protective functions

In general, the proteins encoded by [+/+] memory genes are involved in cell protective roles. These include heat shock proteins and chaperons, proteins involved in the repair of stress-induced damages to membranes, in modifying membrane lipid composition, in regulating membrane fluidity and permeability to toxic ions, dehydrins (RD29B, RAB18, LTi30), and lipid transfer proteins (LTP2, LTP3, LTP4) [26,27]. LEA (late embryogenesis abundant) proteins functioning as molecular chaperones to maintain membrane structures, ion balance and homeostasis [28,29] and enzymes for the synthesis of isoleucine (toxin degradation), serine (redox responses), and proline (an osmolyte) are encoded by [+/+] memory genes. Thereby, [+/+] memory genes ensure elevated synthesis of factors critical for cell survival under multiple dehydration stresses (Table 2; Figure 3).

Abscisic acid (ABA) is the key mediator of dehydration signaling and is involved in responses to other abiotic stress response pathways [30,31]. ABA responsive genes that respond to dehydration are often points of overlap in various abiotic stress pathways [32,33] (Table 2). The [+/+] memory and [+/=] non-memory genes, generally, encode proteins with similar protective functions. However, the memory component, presumably, allows the plant to optimize its stress tolerance within the context of protection. Only a few [-/-] memory genes and a smaller number of down-regulated [-/=] genes are shared between dehydration/ABA and other abiotic stress responding systems (Table 2; Additional file 5: Table S5; Figure 3).

### Photosynthesis and growth during repeated stresses

More than 40% of the [-/-] memory genes encode ribosome/protein synthesis, chloroplast, and thylakoid membrane related proteins indicating repetitive stress results in a stronger attenuation of the pathways for photosystem II electron transport, small RuBisCo subunit and sugar biosynthesis, as well as for ribosome assembly, protein translation, DNA replication and histones (Additional file 4: Table S4; Additional file 5: Table S5; Figure 3). None of the [+/+] memory genes encodes a thylakoid membrane function. Of note, 12 [+/+] memory genes associated with chloroplasts encode functions that are different, even opposite to, the functions encoded by the [-/-] memory genes. Chlorophyll and toxin catabolic enzymes, enzymes for the synthesis of isoleucine (toxin degradation), serine (redox responses), and proline (an osmolyte) are encoded by [+/+] memory genes. Photosynthetic and starch synthesizing activities, in addition to proteins involved in chloroplast organization and relocation, are encoded by [-/-] memory genes. Down-regulated [-/=] non-memory genes encode ribosome- and chloroplast-associated proteins as well (Table 2; Additional file 5: Table S3; Figure 3). The functional implications of the [-/-] transcriptional response patterns (decreased protein synthesis, cell growth and photosynthesis) and the protective functions by the [+/+] memory genes are in agreement with known responses of plants enduring dehydration stress [34-37]. Of note, on the background of a rather even distribution of GO functions encoded by the late responding [=/+] and [=/-] genes, the fraction of genes encoding chloroplast and thylakoid membrane proteins constitute ~ 20% among the [=/-] genes (Table 2; Additional file 5: Table S5).

### Re-adjusting cellular homeostasis

The [-/+] and [+/-] revised memory genes respond robustly to the initial stress, but in a subsequent exposure provide responses at levels closer to their non-stressed levels. Presumably, these genes help in restoring homeostasis as the plant adjusts to dehydration stress. Thus, [-/+] memory genes for chloroplast organization, relocation, photosynthetic, light harvesting, and metabolic functions are similar to the functions encoded by the [-/-] memory genes (Additional file 5: Table S5; Figure 3). However, by producing significantly more transcripts in S3 than in S1([-/+]), or returning to pre-stressed (W) levels in S3 ([+/-]), these genes support the ability of plants to restore photosynthesis after temporary water withdrawal [38]. Presumably, the [**-/+**] genes contribute to an initial decrease in chloroplast/photosynthetic activity in S1, but by reverting to pre-stressed transcription levels in S3, contribute to a subsequent re-adjustment. The [**-/+**] memory genes *GLK1* and *GLK2,* implicated in regulating the photosynthetic apparatus and chloroplast development in a cell-autonomous manner [39,40] are candidate genes for a role in such an adjustment.

Within the same GO category, [-/+] and [+/-] memory genes may encode opposing biochemical activities. For example, a number of the [-/+] memory genes encode chlorophyll and carbohydrate (starch) synthesizing enzymes, while chlorophyll and starch degrading activities are encoded by [+/-] memory genes (Additional file 5: Table S5). Juxtaposing these functions would suggest complementary roles during both the initial and repeated stresses. Acting together, these genes are likely to decrease overall chlorophyll and starch biosynthesis during the first stress but to partially restore it during subsequent stresses. These complementary transcriptional patterns and functional roles support the fine coordination occurring between metabolic and energy adjustments during adaptation to drought [41].

Membrane-associated memory genes present another paradigm of biological relevance for the dehydration memory genes. As dehydration may damage membrane integrity, it is not surprising that a large number of genes (more than 750 of memory and non-memory) encode functions related to membranes; more than 260 of the late responding genes encode membrane-associated proteins (Table 2; Additional file 5: Table S5; Figure 3). Proteins regulating osmotic pressure, water balance, and wall modifications have been implicated in plants’ stress responses and environmental adaptation [42]. These functions are encoded by genes of all four-memory response types, suggesting that after providing an up/down transcriptional response in S1, a large number of Arabidopsis genes alter their transcript outputs (further up- or down-regulating or reversing towards pre-stressed levels) in a subsequent exposure to more finely modify cell membrane structure, ion balance and homeostasis.

### Dehydration stress memory genes in crosstalk with other response pathways

Individual genes that are regulated by multiple different stimuli represent overlapping points of these plant’s signaling networks [43-48]. The subset of the dehydration stress memory genes that are at these convergent points revealed new aspects of plants’ stress responses. For example the dehydration/JA pathways share the largest number of both memory and non-memory genes co-regulated by these pathways, consistent with reported crosstalk between jasmonic acid (JA) and ABA regulated signaling networks during dehydration [45,47,49], salt [50], and cold stresses [51]. More than 200 JA-responsive genes are up-regulated in S1 (including memory and non-memory genes) and about 80 are down-regulated (Table 2; Additional file 5: Table S5). In S3 stress, however, 89 [+/=] and 46 [-/=] genes continued to provide similar transcript levels, seven produced higher ([+/+]), and 16 lower ([-/-]), transcript levels, but 140 ([+/-]) genes did not respond in the subsequent stress. Thereby, in addition to a finer tuning, the altered transcript levels from the memory genes suggest an altered crosstalk between these pathways in S3 compared to S1.

Non-memory ([+/=] and [-/=]) and unidirectional memory ([+/+] and [-/-]) dehydration stress responding genes respond every time the plant experiences dehydration stress. Concurrent with amplified responses from the [+/+] and [-/-] memory genes, however, engagement of the [+/-] and [-/+] genes lessens in S3, suggesting the nature and the dynamics of the interactions between dehydration and other signaling pathways are different during repeated exposures than the interactions occurring in a single exposure.

Lastly, we note that TF encoded by memory genes can be critical for the expression of dependent genes in S1, but that they do not necessarily determine the memory behavior of regulated genes in S3. For example, the [+/-] memory of the *MYC2* gene, identified as a master regulator of the crosstalk between the ABA, SA, GA, JA, and auxin signaling pathways [52-54], correlates with the [+/-] memory of a large number of MYC2-dependent genes common for these signaling pathways (Figure 4C; Additional file 6: Table S6). However, the signature gene, *RD22*, directly regulated by MYC2 [55,56] is a non-memory gene that is up-regulated in both S1 and in S3 (Additional file 4: Table S4; Additional file 6: Table S6) despite the fairly low levels of *MYC2* transcripts in S3. Evidently, another TF activates *RD22* in S3. The results suggest that diverse gene-specific mechanisms are involved in regulating the behavior of dehydration stress responding genes and that the memory behavior of individual TFs alone does not necessarily determine or predict the memory or non-memory behavior of their targets.

## Conclusion

The genome-wide response of Arabidopsis genes to dehydration stress revealed the existence of four distinct transcriptional memory response patterns. By altering transcript levels, and presumably the levels of encoded proteins, memory genes are likely to alter the cellular responses and the crosstalk between overlapping pathways. Adjustments of expression of memory genes, together with the consistent responses from the non-memory genes, allow the plant to optimize its responses and the interactions between various signaling systems. Transcriptional memory, like defense gene priming, can provide the benefits of a more robust or modified stress response while reducing the costs of the state of preparedness [25]. The behavior of transcriptional memory genes adds a new dimension to our understanding of plants’ responses to dehydration stress and to current models for interactions between different signaling systems. Revealing the molecular mechanisms of transcriptional memory responses may be critical for understanding how plants’ adapt to changing environments and is emerging as a new area in plant abiotic and biotic stress response research.

## Authors’ contributions

YD, NL and LV performed experiments, J-JR performed bioinformatics analyses. MF and ZA conceived the study and interpreted results. ZA wrote the paper. All authors read and approved the final manuscript.

## Supplementary Material

Additional file 1: Table S1 Primers used in the qRT-PCR experiments. Click here for file

Additional file 2: Table S2 Distribution of raw and mapped reads over samples and replicates. Click here for file

Additional file 3: Table S3 Transcript abundance established in W, S1 and S3 for all 33, 555 genes of *A. thaliana.* Click here for file

Additional file 4: Table S4 Transcript abundances displayed by the memory genes from the four memory categories and for the induced and repressed non-memory genes of *A. thaliana.* Click here for file

Additional file 5: Table S5 Complete list of memory and non-memory genes according to GO function. Click here for file

Additional file 6: Table S6 Memory and non-memory genes encoding TFs. Click here for file
