# Supplementary material for: Four distinct types of dehydration stress memory genes in Arabidopsis thaliana
Source: BMC Plant Biol. 2013 Dec 30;13:229. doi: 10.1186/1471-2229-13-229 (PMC3879431; doi:10.1186/1471-2229-13-229)
Supplement: Additional file 1: Table S1 — Primers used in the qRT-PCR experiments. [file 1471-2229-13-229-S1.pdf]

Table S1: List of primers used for qRT-PCR analysis

Genes	Primer Name	Sequence (5'-->3')
At3g50970	N327	GGGACTAACACGGCTTATGG
	N328	CCTGGCAGTTGCTCTTTAAT
At1g54160	N266	CCGTGCTAAACTCGAAGCTC
	N267	AGCATGAAGATGGCGAGACT
AT4g34000	N229	AACCGTTCTCAACCTGCAAC
	N230	GCTGCAACCGTTACTCCTGT
At5g28770	N181	CGGTCCAGAAGAAGAAAGCA
	N182	TGAGACCCTTCATGAGTTTTGA
At3g22120	N143	TTCCCTCGTACGTTAATAGTGG
	N144	GGGTTTGTCACACTTTCCTCA
At2g45180	N177	CTCCTAAACCCCCAAAAACC
	N178	TGGTGGAGAACCAACAACAA
At3g54500	Y873	TCTCATCAGGTGCCTGTAGC
	Y874	GCCAGAATCATCTTGTTCCA
At5g45820	N175	AAAGCCGATGTTTGGTCTTG
	N176	ATTTGAATTCGCCTTTCGTG
At1g71030	N171	CATCCTCAAGCTTCATGCAC
	N172	AACTTCGTTGTCGGTTCGTC
At4g28140	N261	CACAGCGCAACACTGAATTT
	N262	TGGCGTTTCAGGTTCTTTCT
At1g19180	N211	GCATGCAAGCCTGATGTCAAT
	N212	TGCCTAGGAAACAGATTCGTC
At1g51780	N249	ACATTCCGAGCCCTTTTACC
	N250	GCTTGCTTGCGTTGTGATAA
At2g42540/COR15A	Y31	CAGATGGTGAGAAAGCGAAA
	Y32	CCCTACTTTGTGGCATCCTT
At4g27410	Y33	AGTTCGATCCTTGGGATTTG
	Y34	ACCCGTTGCTTTCCAATAAC
At3g11020	Y41	TGGTTTAACGGTGGCTGATA
	Y42	TGGTCCTCCTTTACCCTTCA
At4g38770	N179	GCCGAGAGCAAGATCAAAAC
	N180	GTCGTCAATGTTTCCCGAAC
At5g01410	N183	ACCCTGAGATGCTTGTGGAG
	N184	AGCGATTAGCGAACCTCTCA
At4g39260	N349	CGGCGACGTTATCGATTCTA
	N350	TCCTTGAAGGTGACGAATCC
