# Supplementary material for: Four distinct types of dehydration stress memory genes in Arabidopsis thaliana
Source: BMC Plant Biol. 2013 Dec 30;13:229. doi: 10.1186/1471-2229-13-229 (PMC3879431; doi:10.1186/1471-2229-13-229)
Supplement: Additional file 2: Table S2 — Distribution of raw and mapped reads over samples and replicates. [file 1471-2229-13-229-S2.pdf]

Table S2 Distribution of raw and mapped reads over samples and replicates.

Sample NAMe	Raw reads	Mapped reads
S1 REPlicate 1	36614842	34311218 (94 %)
S1 REPlicate 2	40193946	38718225 (96 %)
S3 replicate 1	37706035	35241847 (93 %)
S3 replicate 2	40718457	39328849 (97 %)
Water repLICATE 1	24832092	23001565 (93 %)
Water repLICATE 2	28601911	26515658 (93 %)
